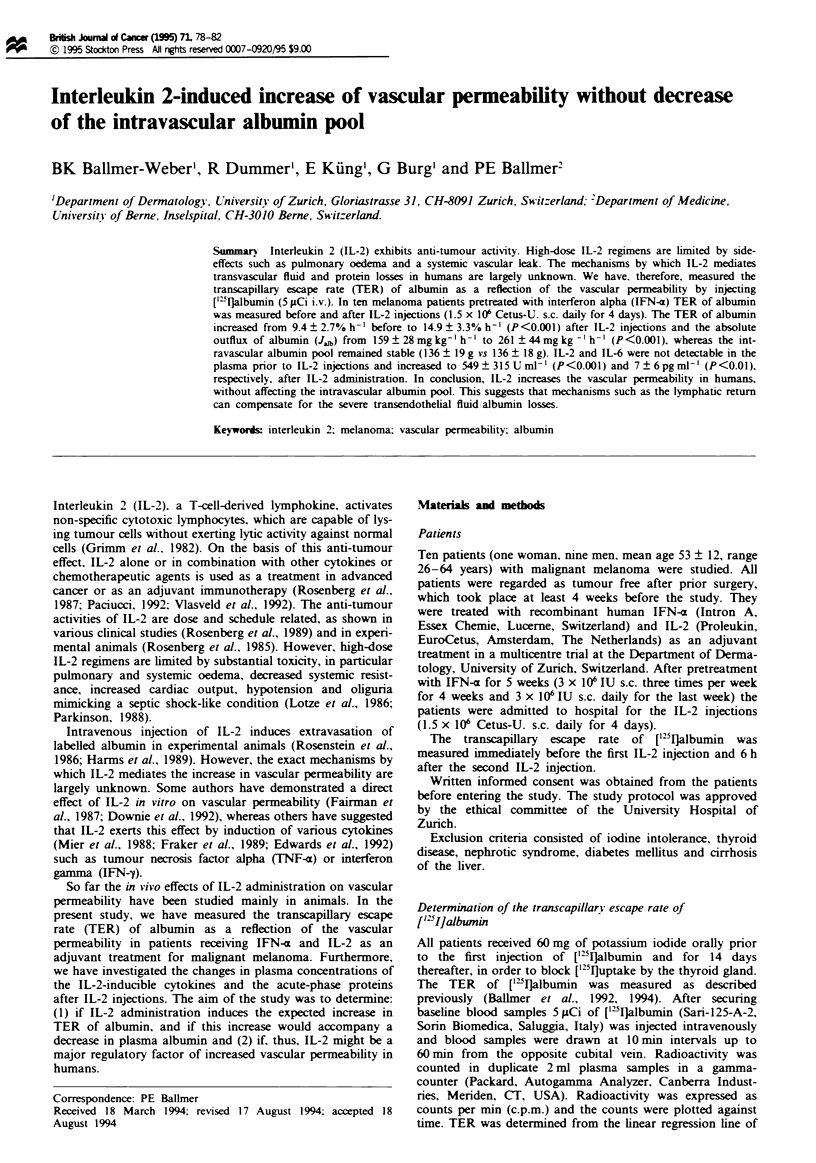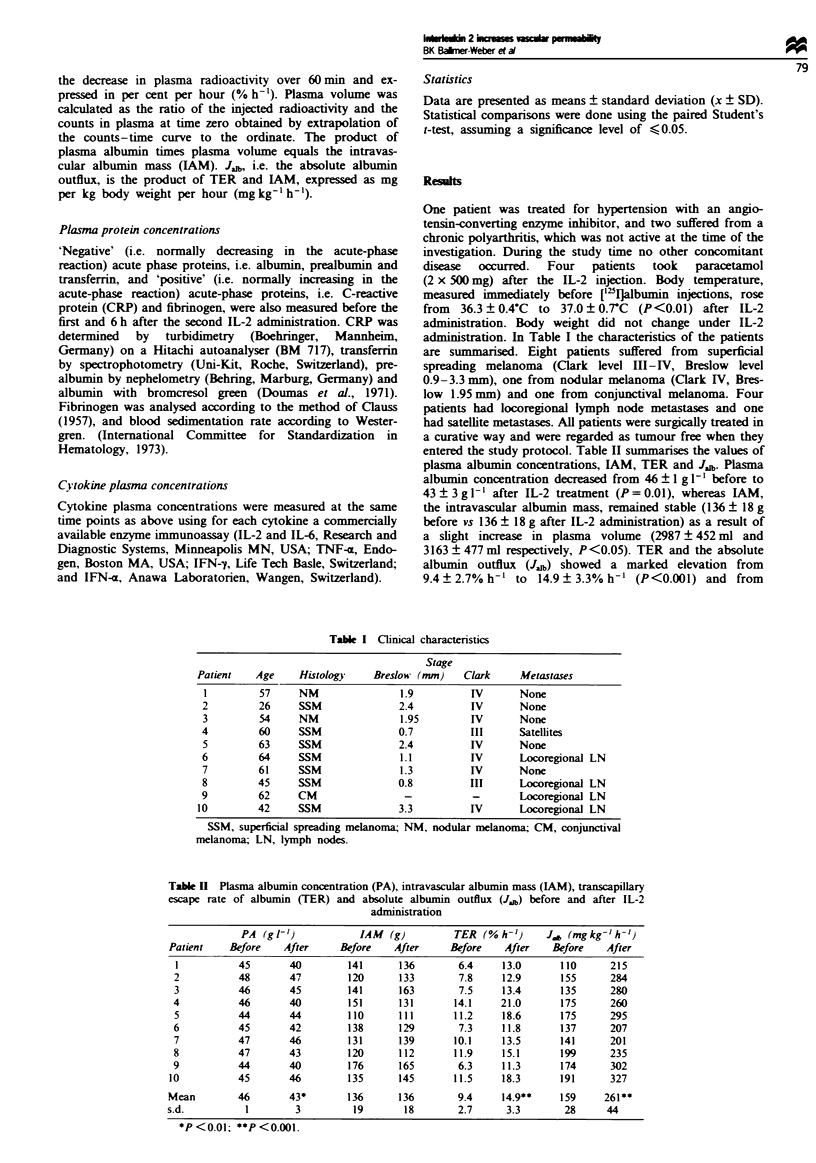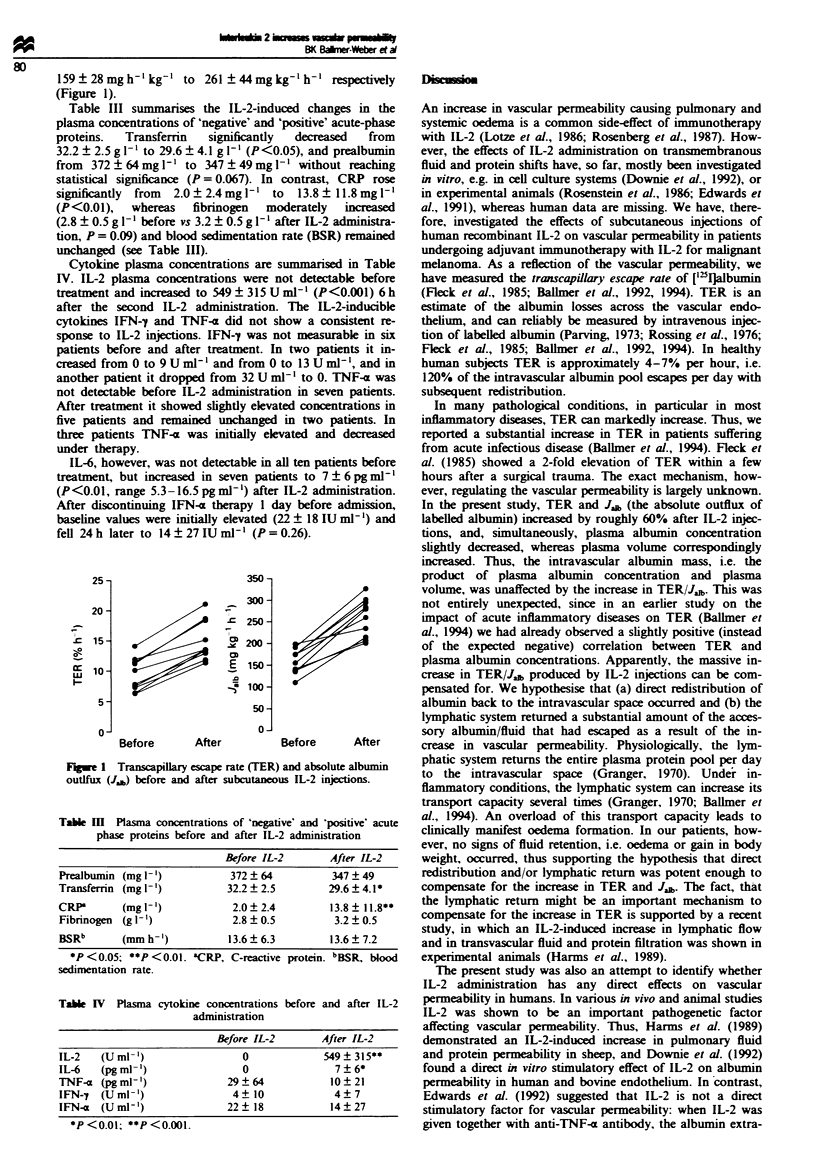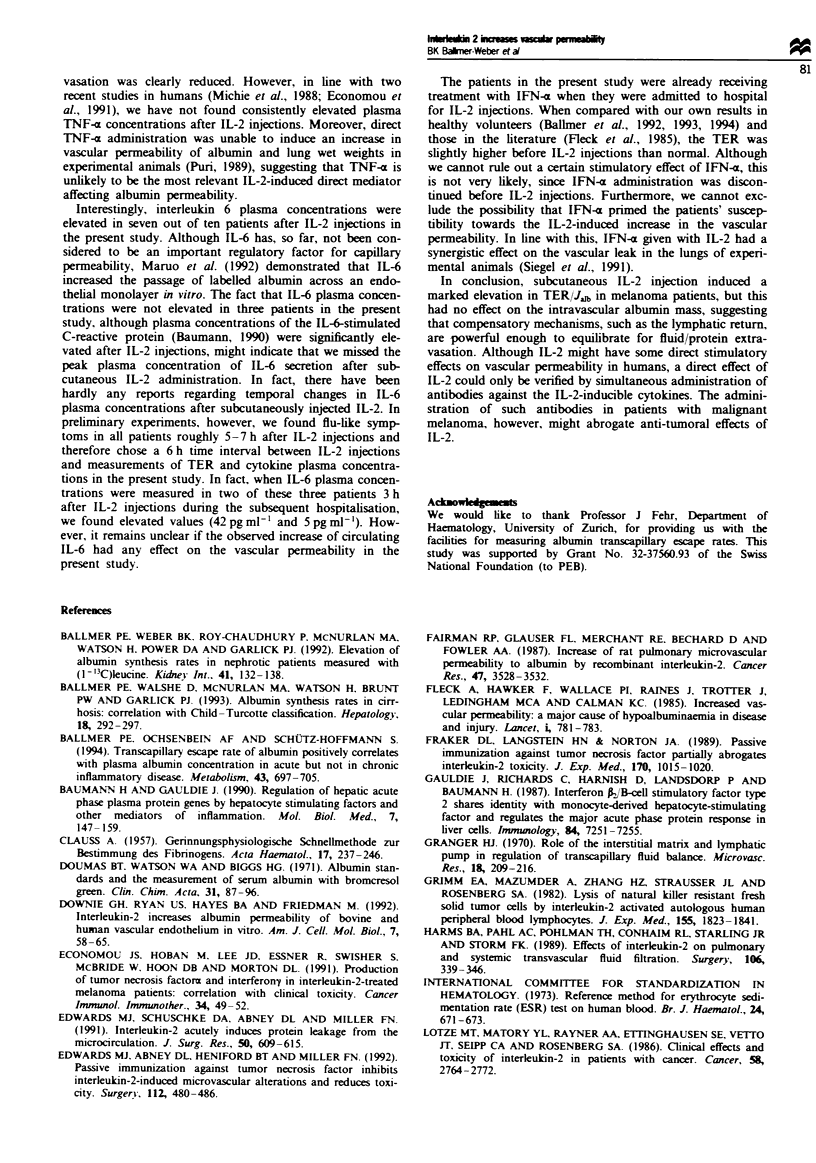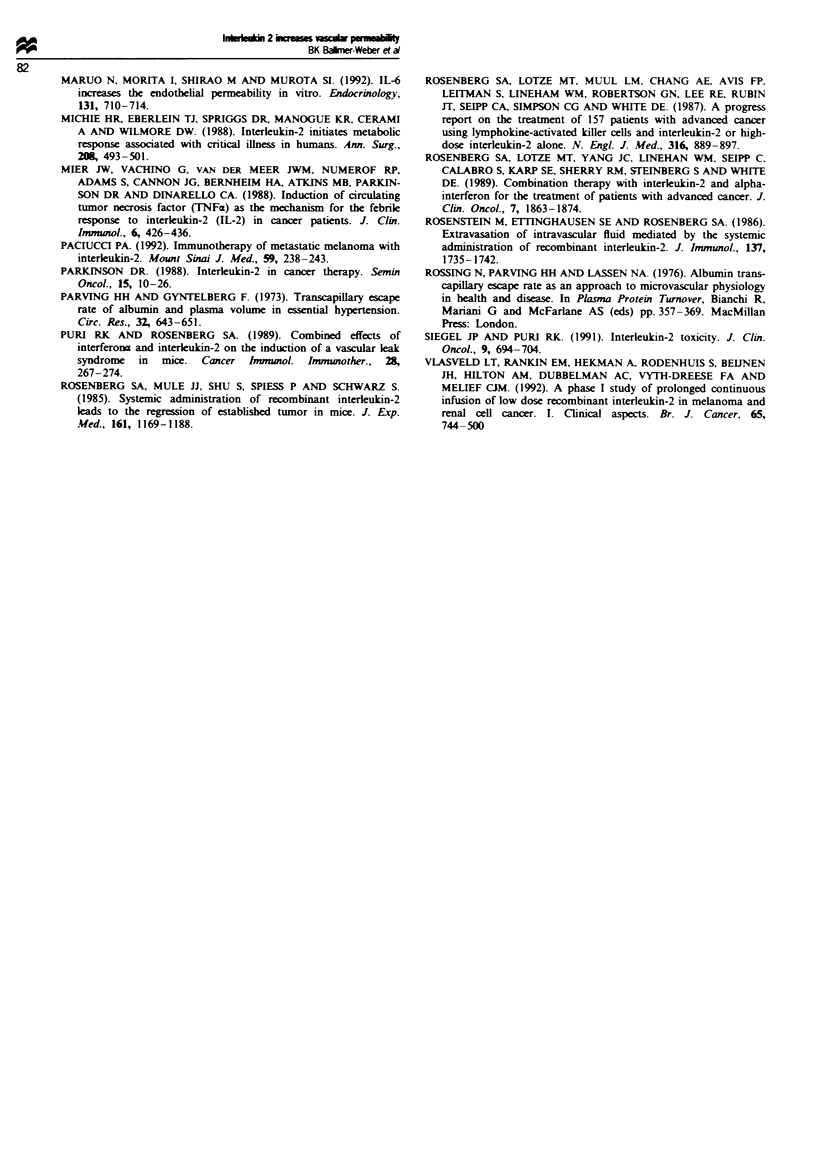# Interleukin 2-induced increase of vascular permeability without decrease of the intravascular albumin pool.

**DOI:** 10.1038/bjc.1995.16

**Published:** 1995-01

**Authors:** B. K. Ballmer-Weber, R. Dummer, E. Küng, G. Burg, P. E. Ballmer

**Affiliations:** Department of Dermatology, University of Zurich, Switzerland.

## Abstract

Interleukin 2 (IL-2) exhibits anti-tumour activity. High-dose IL-2 regimens are limited by side-effects such as pulmonary oedema and a systemic vascular leak. The mechanisms by which IL-2 mediates transvascular fluid and protein losses in humans are largely unknown. We have, therefore, measured the transcapillary escape rate (TER) of albumin as a reflection of the vascular permeability by injecting [125I]albumin (5 microCi i.v.). In ten melanoma patients pretreated with interferon alpha (IFN-alpha) TER of albumin was measured before and after IL-2 injections (1.5 x 10(6) Cetus-U. s.c. daily for 4 days). The TER of albumin increased from 9.4 +/- 2.7% h-1 before to 14.9 +/- 3.3% h-1 (P < 0.001) after IL-2 injections and the absolute outflux of albumin (Jalb) from 159 +/- 28 mg kg-1 h-1 to 261 +/- 44 mg kg-1 h-1 (P < 0.001), whereas the intravascular albumin pool remained stable (136 +/- 19 g vs 136 +/- 18 g). IL-2 and IL-6 were not detectable in the plasma prior to IL-2 injections and increased to 549 +/- 315 U ml-1 (P < 0.001) and 7 +/- 6 pg ml-1 (P < 0.01), respectively, after IL-2 administration. In conclusion, IL-2 increases the vascular permeability in humans, without affecting the intravascular albumin pool. This suggests that mechanisms such as the lymphatic return can compensate for the severe transendothelial fluid/albumin losses.


British Journal of Cancer (1995) 71, 78-82

© 1995 Stockton Press All rights reserved 0007-0920/95 $9.00

### Interleukin 2-induced increase of vascular permeability without decrease of the intravascular albumin pool

BK Ballmer-Weber[1], R Dummer[1], E Küng[1], G Burg[1] and PE Ballmer[2]

[1]*Department of Dermatology, University of Zurich, Gloriastrasse 31, CH-8091 Zurich, Switzerland;* [2]*Department of Medicine, University of Berne, Inselspital, CH-3010 Berne, Switzerland.*

**Summary** Interleukin 2 (IL-2) exhibits anti-tumour activity. High-dose IL-2 regimens are limited by side-effects such as pulmonary oedema and a systemic vascular leak. The mechanisms by which IL-2 mediates transvascular fluid and protein losses in humans are largely unknown. We have, therefore, measured the transcapillary escape rate (TER) of albumin as a reflection of the vascular permeability by injecting [$^{125}$I]albumin (5 $\mu$Ci i.v.). In ten melanoma patients pretreated with interferon alpha (IFN-$\alpha$) TER of albumin was measured before and after IL-2 injections (1.5 × $10^6$ Cetus-U. s.c. daily for 4 days). The TER of albumin increased from 9.4 ± 2.7% $h^{-1}$ before to 14.9 ± 3.3% $h^{-1}$ ($P<0.001$) after IL-2 injections and the absolute outflux of albumin ($J_{alb}$) from 159 ± 28 mg $kg^{-1}$ $h^{-1}$ to 261 ± 44 mg $kg^{-1}$ $h^{-1}$ ($P<0.001$), whereas the intravascular albumin pool remained stable (136 ± 19 g *vs* 136 ± 18 g). IL-2 and IL-6 were not detectable in the plasma prior to IL-2 injections and increased to 549 ± 315 U $ml^{-1}$ ($P<0.001$) and 7 ± 6 pg $ml^{-1}$ ($P<0.01$), respectively, after IL-2 administration. In conclusion, IL-2 increases the vascular permeability in humans, without affecting the intravascular albumin pool. This suggests that mechanisms such as the lymphatic return can compensate for the severe transendothelial fluid/albumin losses.

**Keywords:** interleukin 2; melanoma; vascular permeability; albumin

Interleukin 2 (IL-2), a T-cell-derived lymphokine, activates non-specific cytotoxic lymphocytes, which are capable of lysing tumour cells without exerting lytic activity against normal cells (Grimm *et al.*, 1982). On the basis of this anti-tumour effect, IL-2 alone or in combination with other cytokines or chemotherapeutic agents is used as a treatment in advanced cancer or as an adjuvant immunotherapy (Rosenberg *et al.*, 1987; Paciucci, 1992; Vlasveld *et al.*, 1992). The anti-tumour activities of IL-2 are dose and schedule related, as shown in various clinical studies (Rosenberg *et al.*, 1989) and in experimental animals (Rosenberg *et al.*, 1985). However, high-dose IL-2 regimens are limited by substantial toxicity, in particular pulmonary and systemic oedema, decreased systemic resistance, increased cardiac output, hypotension and oliguria mimicking a septic shock-like condition (Lotze *et al.*, 1986; Parkinson, 1988).

Intravenous injection of IL-2 induces extravasation of labelled albumin in experimental animals (Rosenstein *et al.*, 1986; Harms *et al.*, 1989). However, the exact mechanisms by which IL-2 mediates the increase in vascular permeability are largely unknown. Some authors have demonstrated a direct effect of IL-2 *in vitro* on vascular permeability (Fairman *et al.*, 1987; Downie *et al.*, 1992), whereas others have suggested that IL-2 exerts this effect by induction of various cytokines (Mier *et al.*, 1988; Fraker *et al.*, 1989; Edwards *et al.*, 1992) such as tumour necrosis factor alpha (TNF-$\alpha$) or interferon gamma (IFN-$\gamma$).

So far the *in vivo* effects of IL-2 administration on vascular permeability have been studied mainly in animals. In the present study, we have measured the transcapillary escape rate (TER) of albumin as a reflection of the vascular permeability in patients receiving IFN-$\alpha$ and IL-2 as an adjuvant treatment for malignant melanoma. Furthermore, we have investigated the changes in plasma concentrations of the IL-2-inducible cytokines and the acute-phase proteins after IL-2 injections. The aim of the study was to determine: (1) if IL-2 administration induces the expected increase in TER of albumin, and if this increase would accompany a decrease in plasma albumin and (2) if, thus, IL-2 might be a major regulatory factor of increased vascular permeability in humans.

Correspondence: PE Ballmer
Received 18 March 1994; revised 17 August 1994; accepted 18 August 1994

### Materials and methods

### *Patients*

Ten patients (one woman, nine men, mean age 53 ± 12, range 26–64 years) with malignant melanoma were studied. All patients were regarded as tumour free after prior surgery, which took place at least 4 weeks before the study. They were treated with recombinant human IFN-$\alpha$ (Intron A, Essex Chemie, Lucerne, Switzerland) and IL-2 (Proleukin, EuroCetus, Amsterdam, The Netherlands) as an adjuvant treatment in a multicentre trial at the Department of Dermatology, University of Zurich, Switzerland. After pretreatment with IFN-$\alpha$ for 5 weeks (3 × $10^6$ IU s.c. three times per week for 4 weeks and 3 × $10^6$ IU s.c. daily for the last week) the patients were admitted to hospital for the IL-2 injections (1.5 × $10^6$ Cetus-U. s.c. daily for 4 days).

The transcapillary escape rate of [$^{125}$I]albumin was measured immediately before the first IL-2 injection and 6 h after the second IL-2 injection.

Written informed consent was obtained from the patients before entering the study. The study protocol was approved by the ethical committee of the University Hospital of Zurich.

Exclusion criteria consisted of iodine intolerance, thyroid disease, nephrotic syndrome, diabetes mellitus and cirrhosis of the liver.

### *Determination of the transcapillary escape rate of [$^{125}$I]albumin*

All patients received 60 mg of potassium iodide orally prior to the first injection of [$^{125}$I]albumin and for 14 days thereafter, in order to block [$^{125}$I]uptake by the thyroid gland. The TER of [$^{125}$I]albumin was measured as described previously (Ballmer *et al.*, 1992, 1994). After securing baseline blood samples 5 $\mu$Ci of [$^{125}$I]albumin (Sari-125-A-2, Sorin Biomedica, Saluggia, Italy) was injected intravenously and blood samples were drawn at 10 min intervals up to 60 min from the opposite cubital vein. Radioactivity was counted in duplicate 2 ml plasma samples in a gamma-counter (Packard, Autogamma Analyzer, Canberra Industries, Meriden, CT, USA). Radioactivity was expressed as counts per min (c.p.m.) and the counts were plotted against time. TER was determined from the linear regression line of the decrease in plasma radioactivity over 60 min and expressed in per cent per hour (% $h^{-1}$). Plasma volume was calculated as the ratio of the injected radioactivity and the counts in plasma at time zero obtained by extrapolation of the counts-time curve to the ordinate. The product of plasma albumin times plasma volume equals the intravascular albumin mass (IAM). $J_{alb}$, i.e. the absolute albumin outflux, is the product of TER and IAM, expressed as mg per kg body weight per hour (mg $kg^{-1}$ $h^{-1}$).

*Plasma protein concentrations*

'Negative' (i.e. normally decreasing in the acute-phase reaction) acute phase proteins, i.e. albumin, prealbumin and transferrin, and 'positive' (i.e. normally increasing in the acute-phase reaction) acute-phase proteins, i.e. C-reactive protein (CRP) and fibrinogen, were also measured before the first and 6 h after the second IL-2 administration. CRP was determined by turbidimetry (Boehringer, Mannheim, Germany) on a Hitachi autoanalyser (BM 717), transferrin by spectrophotometry (Uni-Kit, Roche, Switzerland), prealbumin by nephelometry (Behring, Marburg, Germany) and albumin with bromcresol green (Doumas *et al.*, 1971). Fibrinogen was analysed according to the method of Clauss (1957), and blood sedimentation rate according to Westergren. (International Committee for Standardization in Hematology, 1973).

*Cytokine plasma concentrations*

Cytokine plasma concentrations were measured at the same time points as above using for each cytokine a commercially available enzyme immunoassay (IL-2 and IL-6, Research and Diagnostic Systems, Minneapolis MN, USA; TNF-α, Endogen, Boston MA, USA; IFN-γ, Life Tech Basle, Switzerland; and IFN-α, Anawa Laboratorien, Wangen, Switzerland).

*Statistics*

Data are presented as means ± standard deviation ($x \pm$ SD). Statistical comparisons were done using the paired Student's *t*-test, assuming a significance level of $\leqslant 0.05$.

### Results

One patient was treated for hypertension with an angiotensin-converting enzyme inhibitor, and two suffered from a chronic polyarthritis, which was not active at the time of the investigation. During the study time no other concomitant disease occurred. Four patients took paracetamol (2 x 500 mg) after the IL-2 injection. Body temperature, measured immediately before [$^{125}$I]albumin injections, rose from 36.3 ± 0.4°C to 37.0 ± 0.7°C ($P<0.01$) after IL-2 administration. Body weight did not change under IL-2 administration. In Table I the characteristics of the patients are summarised. Eight patients suffered from superficial spreading melanoma (Clark level III-IV, Breslow level 0.9-3.3 mm), one from nodular melanoma (Clark IV, Breslow 1.95 mm) and one from conjunctival melanoma. Four patients had locoregional lymph node metastases and one had satellite metastases. All patients were surgically treated in a curative way and were regarded as tumour free when they entered the study protocol. Table II summarises the values of plasma albumin concentrations, IAM, TER and $J_{alb}$. Plasma albumin concentration decreased from 46 ± 1 g $l^{-1}$ before to 43 ± 3 g $l^{-1}$ after IL-2 treatment ($P = 0.01$), whereas IAM, the intravascular albumin mass, remained stable (136 ± 18 g before *vs* 136 ± 18 g after IL-2 administration) as a result of a slight increase in plasma volume (2987 ± 452 ml and 3163 ± 477 ml respectively, $P<0.05$). TER and the absolute albumin outflux ($J_{alb}$) showed a marked elevation from 9.4 ± 2.7% $h^{-1}$ to 14.9 ± 3.3% $h^{-1}$ ($P<0.001$) and from

**Table I** Clinical characteristics

| Patient | Age | Histology | Stage: Breslow (mm) | Stage: Clark | Metastases |
|---|---|---|---|---|---|
| 1 | 57 | NM | 1.9 | IV | None |
| 2 | 26 | SSM | 2.4 | IV | None |
| 3 | 54 | NM | 1.95 | IV | None |
| 4 | 60 | SSM | 0.7 | III | Satellites |
| 5 | 63 | SSM | 2.4 | IV | None |
| 6 | 64 | SSM | 1.1 | IV | Locoregional LN |
| 7 | 61 | SSM | 1.3 | IV | None |
| 8 | 45 | SSM | 0.8 | III | Locoregional LN |
| 9 | 62 | CM | – | – | Locoregional LN |
| 10 | 42 | SSM | 3.3 | IV | Locoregional LN |

SSM, superficial spreading melanoma; NM, nodular melanoma; CM, conjunctival melanoma; LN, lymph nodes.

**Table II** Plasma albumin concentration (PA), intravascular albumin mass (IAM), transcapillary escape rate of albumin (TER) and absolute albumin outflux ($J_{alb}$) before and after IL-2 administration

| Patient | PA (g $l^{-1}$) Before | PA After | IAM (g) Before | IAM After | TER (% $h^{-1}$) Before | TER After | $J_{alb}$ (mg $kg^{-1}$ $h^{-1}$) Before | $J_{alb}$ After |
|---|---|---|---|---|---|---|---|---|
| 1 | 45 | 40 | 141 | 136 | 6.4 | 13.0 | 110 | 215 |
| 2 | 48 | 47 | 120 | 133 | 7.8 | 12.9 | 155 | 284 |
| 3 | 46 | 45 | 141 | 163 | 7.5 | 13.4 | 135 | 280 |
| 4 | 46 | 40 | 151 | 131 | 14.1 | 21.0 | 175 | 260 |
| 5 | 44 | 44 | 110 | 111 | 11.2 | 18.6 | 175 | 295 |
| 6 | 45 | 42 | 138 | 129 | 7.3 | 11.8 | 137 | 207 |
| 7 | 47 | 46 | 131 | 139 | 10.1 | 13.5 | 141 | 201 |
| 8 | 47 | 43 | 120 | 112 | 11.9 | 15.1 | 199 | 235 |
| 9 | 44 | 40 | 176 | 165 | 6.3 | 11.3 | 174 | 302 |
| 10 | 45 | 46 | 135 | 145 | 11.5 | 18.3 | 191 | 327 |
| Mean | 46 | 43* | 136 | 136 | 9.4 | 14.9** | 159 | 261** |
| s.d. | 1 | 3 | 19 | 18 | 2.7 | 3.3 | 28 | 44 |

*$P<0.01$; **$P<0.001$.

$159 \pm 28$ mg h$^{-1}$ kg$^{-1}$ to $261 \pm 44$ mg kg$^{-1}$ h$^{-1}$ respectively (Figure 1).

Table III summarises the IL-2-induced changes in the plasma concentrations of 'negative' and 'positive' acute-phase proteins. Transferrin significantly decreased from $32.2 \pm 2.5$ g l$^{-1}$ to $29.6 \pm 4.1$ g l$^{-1}$ ($P<0.05$), and prealbumin from $372 \pm 64$ mg l$^{-1}$ to $347 \pm 49$ mg l$^{-1}$ without reaching statistical significance ($P = 0.067$). In contrast, CRP rose significantly from $2.0 \pm 2.4$ mg l$^{-1}$ to $13.8 \pm 11.8$ mg l$^{-1}$ ($P<0.01$), whereas fibrinogen moderately increased ($2.8 \pm 0.5$ g l$^{-1}$ before *vs* $3.2 \pm 0.5$ g l$^{-1}$ after IL-2 administration, $P = 0.09$) and blood sedimentation rate (BSR) remained unchanged (see Table III).

Cytokine plasma concentrations are summarised in Table IV. IL-2 plasma concentrations were not detectable before treatment and increased to $549 \pm 315$ U ml$^{-1}$ ($P<0.001$) 6 h after the second IL-2 administration. The IL-2-inducible cytokines IFN-γ and TNF-α did not show a consistent response to IL-2 injections. IFN-γ was not measurable in six patients before and after treatment. In two patients it increased from 0 to 9 U ml$^{-1}$ and from 0 to 13 U ml$^{-1}$, and in another patient it dropped from 32 U ml$^{-1}$ to 0. TNF-α was not detectable before IL-2 administration in seven patients. After treatment it showed slightly elevated concentrations in five patients and remained unchanged in two patients. In three patients TNF-α was initially elevated and decreased under therapy.

IL-6, however, was not detectable in all ten patients before treatment, but increased in seven patients to $7 \pm 6$ pg ml$^{-1}$ ($P<0.01$, range 5.3–16.5 pg ml$^{-1}$) after IL-2 administration. After discontinuing IFN-α therapy 1 day before admission, baseline values were initially elevated ($22 \pm 18$ IU ml$^{-1}$) and fell 24 h later to $14 \pm 27$ IU ml$^{-1}$ ($P = 0.26$).

**Figure 1** Transcapillary escape rate (TER) and absolute albumin outlfux ($J_{alb}$) before and after subcutaneous IL-2 injections.

**Table III** Plasma concentrations of 'negative' and 'positive' acute phase proteins before and after IL-2 administration

| | | *Before IL-2* | *After IL-2* |
|---|---|---|---|
| Prealbumin | (mg l$^{-1}$) | $372 \pm 64$ | $347 \pm 49$ |
| Transferrin | (mg l$^{-1}$) | $32.2 \pm 2.5$ | $29.6 \pm 4.1$* |
| CRP[a] | (mg l$^{-1}$) | $2.0 \pm 2.4$ | $13.8 \pm 11.8$** |
| Fibrinogen | (g l$^{-1}$) | $2.8 \pm 0.5$ | $3.2 \pm 0.5$ |
| BSR[b] | (mm h$^{-1}$) | $13.6 \pm 6.3$ | $13.6 \pm 7.2$ |

*$P<0.05$; **$P<0.01$. [a]CRP, C-reactive protein. [b]BSR, blood sedimentation rate.

**Table IV** Plasma cytokine concentrations before and after IL-2 administration

| | | *Before IL-2* | *After IL-2* |
|---|---|---|---|
| IL-2 | (U ml$^{-1}$) | 0 | $549 \pm 315$** |
| IL-6 | (pg ml$^{-1}$) | 0 | $7 \pm 6$* |
| TNF-α | (pg ml$^{-1}$) | $29 \pm 64$ | $10 \pm 21$ |
| IFN-γ | (U ml$^{-1}$) | $4 \pm 10$ | $4 \pm 7$ |
| IFN-α | (U ml$^{-1}$) | $22 \pm 18$ | $14 \pm 27$ |

*$P<0.01$; **$P<0.001$.

### Discussion

An increase in vascular permeability causing pulmonary and systemic oedema is a common side-effect of immunotherapy with IL-2 (Lotze *et al.*, 1986; Rosenberg *et al.*, 1987). However, the effects of IL-2 administration on transmembranous fluid and protein shifts have, so far, mostly been investigated *in vitro*, e.g. in cell culture systems (Downie *et al.*, 1992), or in experimental animals (Rosenstein *et al.*, 1986; Edwards *et al.*, 1991), whereas human data are missing. We have, therefore, investigated the effects of subcutaneous injections of human recombinant IL-2 on vascular permeability in patients undergoing adjuvant immunotherapy with IL-2 for malignant melanoma. As a reflection of the vascular permeability, we have measured the *transcapillary escape rate* of [$^{125}$I]albumin (Fleck *et al.*, 1985; Ballmer *et al.*, 1992, 1994). TER is an estimate of the albumin losses across the vascular endothelium, and can reliably be measured by intravenous injection of labelled albumin (Parving, 1973; Rossing *et al.*, 1976; Fleck *et al.*, 1985; Ballmer *et al.*, 1992, 1994). In healthy human subjects TER is approximately 4–7% per hour, i.e. 120% of the intravascular albumin pool escapes per day with subsequent redistribution.

In many pathological conditions, in particular in most inflammatory diseases, TER can markedly increase. Thus, we reported a substantial increase in TER in patients suffering from acute infectious disease (Ballmer *et al.*, 1994). Fleck *et al.* (1985) showed a 2-fold elevation of TER within a few hours after a surgical trauma. The exact mechanism, however, regulating the vascular permeability is largely unknown. In the present study, TER and $J_{alb}$ (the absolute outflux of labelled albumin) increased by roughly 60% after IL-2 injections, and, simultaneously, plasma albumin concentration slightly decreased, whereas plasma volume correspondingly increased. Thus, the intravascular albumin mass, i.e. the product of plasma albumin concentration and plasma volume, was unaffected by the increase in TER/$J_{alb}$. This was not entirely unexpected, since in an earlier study on the impact of acute inflammatory diseases on TER (Ballmer *et al.*, 1994) we had already observed a slightly positive (instead of the expected negative) correlation between TER and plasma albumin concentrations. Apparently, the massive increase in TER/$J_{alb}$ produced by IL-2 injections can be compensated for. We hypothesise that (a) direct redistribution of albumin back to the intravascular space occurred and (b) the lymphatic system returned a substantial amount of the accessory albumin/fluid that had escaped as a result of the increase in vascular permeability. Physiologically, the lymphatic system returns the entire plasma protein pool per day to the intravascular space (Granger, 1970). Under inflammatory conditions, the lymphatic system can increase its transport capacity several times (Granger, 1970; Ballmer *et al.*, 1994). An overload of this transport capacity leads to clinically manifest oedema formation. In our patients, however, no signs of fluid retention, i.e. oedema or gain in body weight, occurred, thus supporting the hypothesis that direct redistribution and/or lymphatic return was potent enough to compensate for the increase in TER and $J_{alb}$. The fact, that the lymphatic return might be an important mechanism to compensate for the increase in TER is supported by a recent study, in which an IL-2-induced increase in lymphatic flow and in transvascular fluid and protein filtration was shown in experimental animals (Harms *et al.*, 1989).

The present study was also an attempt to identify whether IL-2 administration has any direct effects on vascular permeability in humans. In various *in vivo* and animal studies IL-2 was shown to be an important pathogenetic factor affecting vascular permeability. Thus, Harms *et al.* (1989) demonstrated an IL-2-induced increase in pulmonary fluid and protein permeability in sheep, and Downie *et al.* (1992) found a direct *in vitro* stimulatory effect of IL-2 on albumin permeability in human and bovine endothelium. In contrast, Edwards *et al.* (1992) suggested that IL-2 is not a direct stimulatory factor for vascular permeability: when IL-2 was given together with anti-TNF-α antibody, the albumin extravasation was clearly reduced. However, in line with two recent studies in humans (Michie *et al.*, 1988; Economou *et al.*, 1991), we have not found consistently elevated plasma TNF-α concentrations after IL-2 injections. Moreover, direct TNF-α administration was unable to induce an increase in vascular permeability of albumin and lung wet weights in experimental animals (Puri, 1989), suggesting that TNF-α is unlikely to be the most relevant IL-2-induced direct mediator affecting albumin permeability.

Interestingly, interleukin 6 plasma concentrations were elevated in seven out of ten patients after IL-2 injections in the present study. Although IL-6 has, so far, not been considered to be an important regulatory factor for capillary permeability, Maruo *et al.* (1992) demonstrated that IL-6 increased the passage of labelled albumin across an endothelial monolayer *in vitro*. The fact that IL-6 plasma concentrations were not elevated in three patients in the present study, although plasma concentrations of the IL-6-stimulated C-reactive protein (Baumann, 1990) were significantly elevated after IL-2 injections, might indicate that we missed the peak plasma concentration of IL-6 secretion after subcutaneous IL-2 administration. In fact, there have been hardly any reports regarding temporal changes in IL-6 plasma concentrations after subcutaneously injected IL-2. In preliminary experiments, however, we found flu-like symptoms in all patients roughly 5–7 h after IL-2 injections and therefore chose a 6 h time interval between IL-2 injections and measurements of TER and cytokine plasma concentrations in the present study. In fact, when IL-6 plasma concentrations were measured in two of these three patients 3 h after IL-2 injections during the subsequent hospitalisation, we found elevated values (42 pg $ml^{-1}$ and 5 pg $ml^{-1}$). However, it remains unclear if the observed increase of circulating IL-6 had any effect on the vascular permeability in the present study.

The patients in the present study were already receiving treatment with IFN-α when they were admitted to hospital for IL-2 injections. When compared with our own results in healthy volunteers (Ballmer *et al.*, 1992, 1993, 1994) and those in the literature (Fleck *et al.*, 1985), the TER was slightly higher before IL-2 injections than normal. Although we cannot rule out a certain stimulatory effect of IFN-α, this is not very likely, since IFN-α administration was discontinued before IL-2 injections. Furthermore, we cannot exclude the possibility that IFN-α primed the patients' susceptibility towards the IL-2-induced increase in the vascular permeability. In line with this, IFN-α given with IL-2 had a synergistic effect on the vascular leak in the lungs of experimental animals (Siegel *et al.*, 1991).

In conclusion, subcutaneous IL-2 injection induced a marked elevation in $TER/J_{alb}$ in melanoma patients, but this had no effect on the intravascular albumin mass, suggesting that compensatory mechanisms, such as the lymphatic return, are powerful enough to equilibrate for fluid/protein extravasation. Although IL-2 might have some direct stimulatory effects on vascular permeability in humans, a direct effect of IL-2 could only be verified by simultaneous administration of antibodies against the IL-2-inducible cytokines. The administration of such antibodies in patients with malignant melanoma, however, might abrogate anti-tumoral effects of IL-2.

**Acknowledgements**
We would like to thank Professor J Fehr, Department of Haematology, University of Zurich, for providing us with the facilities for measuring albumin transcapillary escape rates. This study was supported by Grant No. 32-37560.93 of the Swiss National Foundation (to PEB).

### References

BALLMER PE, WEBER BK, ROY-CHAUDHURY P, MCNURLAN MA, WATSON H, POWER DA AND GARLICK PJ. (1992). Elevation of albumin synthesis rates in nephrotic patients measured with (1-$^{13}$C)leucine. *Kidney Int.*, **41**, 132–138.

BALLMER PE, WALSHE D, MCNURLAN MA, WATSON H, BRUNT PW AND GARLICK PJ. (1993). Albumin synthesis rates in cirrhosis: correlation with Child–Turcotte classification. *Hepatology*, **18**, 292–297.

BALLMER PE, OCHSENBEIN AF AND SCHÜTZ-HOFFMANN S. (1994). Transcapillary escape rate of albumin positively correlates with plasma albumin concentration in acute but not in chronic inflammatory disease. *Metabolism*, **43**, 697–705.

BAUMANN H AND GAULDIE J. (1990). Regulation of hepatic acute phase plasma protein genes by hepatocyte stimulating factors and other mediators of inflammation. *Mol. Biol. Med.*, **7**, 147–159.

CLAUSS A. (1957). Gerinnungsphysiologische Schnellmethode zur Bestimmung des Fibrinogens. *Acta Haematol.*, **17**, 237–246.

DOUMAS BT, WATSON WA AND BIGGS HG. (1971). Albumin standards and the measurement of serum albumin with bromcresol green. *Clin. Chim. Acta*, **31**, 87–96.

DOWNIE GH, RYAN US, HAYES BA AND FRIEDMAN M. (1992). Interleukin-2 increases albumin permeability of bovine and human vascular endothelium in vitro. *Am. J. Cell. Mol. Biol.*, **7**, 58–65.

ECONOMOU JS, HOBAN M, LEE JD, ESSNER R, SWISHER S, MCBRIDE W, HOON DB AND MORTON DL. (1991). Production of tumor necrosis factorα and interferonγ in interleukin-2-treated melanoma patients: correlation with clinical toxicity. *Cancer Immunol. Immunother.*, **34**, 49–52.

EDWARDS MJ, SCHUSCHKE DA, ABNEY DL AND MILLER FN. (1991). Interleukin-2 acutely induces protein leakage from the microcirculation. *J. Surg. Res.*, **50**, 609–615.

EDWARDS MJ, ABNEY DL, HENIFORD BT AND MILLER FN. (1992). Passive immunization against tumor necrosis factor inhibits interleukin-2-induced microvascular alterations and reduces toxicity. *Surgery*, **112**, 480–486.

FAIRMAN RP, GLAUSER FL, MERCHANT RE, BECHARD D AND FOWLER AA. (1987). Increase of rat pulmonary microvascular permeability to albumin by recombinant interleukin-2. *Cancer Res.*, **47**, 3528–3532.

FLECK A, HAWKER F, WALLACE PI, RAINES J, TROTTER J, LEDINGHAM MCA AND CALMAN KC. (1985). Increased vascular permeability: a major cause of hypoalbuminaemia in disease and injury. *Lancet*, **i**, 781–783.

FRAKER DL, LANGSTEIN HN & NORTON JA. (1989). Passive immunization against tumor necrosis factor partially abrogates interleukin-2 toxicity. *J. Exp. Med.*, **170**, 1015–1020.

GAULDIE J, RICHARDS C, HARNISH D, LANDSDORP P AND BAUMANN H. (1987). Interferon $\beta_2$/B-cell stimulatory factor type 2 shares identity with monocyte-derived hepatocyte-stimulating factor and regulates the major acute phase protein response in liver cells. *Immunology*, **84**, 7251–7255.

GRANGER HJ. (1970). Role of the interstitial matrix and lymphatic pump in regulation of transcapillary fluid balance. *Microvasc. Res.*, **18**, 209–216.

GRIMM EA, MAZUMDER A, ZHANG HZ, STRAUSSER JL AND ROSENBERG SA. (1982). Lysis of natural killer resistant fresh solid tumor cells by interleukin-2 activated autologous human peripheral blood lymphocytes. *J. Exp. Med.*, **155**, 1823–1841.

HARMS BA, PAHL AC, POHLMAN TH, CONHAIM RL, STARLING JR AND STORM FK. (1989). Effects of interleukin-2 on pulmonary and systemic transvascular fluid filtration. *Surgery*, **106**, 339–346.

INTERNATIONAL COMMITTEE FOR STANDARDIZATION IN HEMATOLOGY. (1973). Reference method for erythrocyte sedimentation rate (ESR) test on human blood. *Br. J. Haematol.*, **24**, 671–673.

LOTZE MT, MATORY YL, RAYNER AA, ETTINGHAUSEN SE, VETTO JT, SEIPP CA AND ROSENBERG SA. (1986). Clinical effects and toxicity of interleukin-2 in patients with cancer. *Cancer*, **58**, 2764–2772.

MARUO N, MORITA I, SHIRAO M AND MUROTA SI. (1992). IL-6 increases the endothelial permeability in vitro. *Endocrinology*, **131**, 710-714.

MICHIE HR, EBERLEIN TJ, SPRIGGS DR, MANOGUE KR, CERAMI A AND WILMORE DW. (1988). Interleukin-2 initiates metabolic response associated with critical illness in humans. *Ann. Surg.*, **208**, 493-501.

MIER JW, VACHINO G, VAN DER MEER JWM, NUMEROF RP, ADAMS S, CANNON JG, BERNHEIM HA, ATKINS MB, PARKINSON DR AND DINARELLO CA. (1988). Induction of circulating tumor necrosis factor (TNFα) as the mechanism for the febrile response to interleukin-2 (IL-2) in cancer patients. *J. Clin. Immunol.*, **6**, 426-436.

PACIUCCI PA. (1992). Immunotherapy of metastatic melanoma with interleukin-2. *Mount Sinai J. Med.*, **59**, 238-243.

PARKINSON DR. (1988). Interleukin-2 in cancer therapy. *Semin Oncol.*, **15**, 10-26.

PARVING HH AND GYNTELBERG F. (1973). Transcapillary escape rate of albumin and plasma volume in essential hypertension. *Circ. Res.*, **32**, 643-651.

PURI RK AND ROSENBERG SA. (1989). Combined effects of interferonα and interleukin-2 on the induction of a vascular leak syndrome in mice. *Cancer Immunol. Immunother.*, **28**, 267-274.

ROSENBERG SA, MULE JJ, SHU S, SPIESS P AND SCHWARZ S. (1985). Systemic administration of recombinant interleukin-2 leads to the regression of established tumor in mice. *J. Exp. Med.*, **161**, 1169-1188.

ROSENBERG SA, LOTZE MT, MUUL LM, CHANG AE, AVIS FP, LEITMAN S, LINEHAM WM, ROBERTSON GN, LEE RE, RUBIN JT, SEIPP CA, SIMPSON CG AND WHITE DE. (1987). A progress report on the treatment of 157 patients with advanced cancer using lymphokine-activated killer cells and interleukin-2 or high-dose interleukin-2 alone. *N. Engl. J. Med.*, **316**, 889-897.

ROSENBERG SA, LOTZE MT, YANG JC, LINEHAN WM, SEIPP C, CALABRO S, KARP SE, SHERRY RM, STEINBERG S AND WHITE DE. (1989). Combination therapy with interleukin-2 and alpha-interferon for the treatment of patients with advanced cancer. *J. Clin. Oncol.*, **7**, 1863-1874.

ROSENSTEIN M, ETTINGHAUSEN SE AND ROSENBERG SA. (1986). Extravasation of intravascular fluid mediated by the systemic administration of recombinant interleukin-2. *J. Immunol.*, **137**, 1735-1742.

ROSSING N, PARVING HH AND LASSEN NA. (1976). Albumin transcapillary escape rate as an approach to microvascular physiology in health and disease. In *Plasma Protein Turnover*, Bianchi R, Mariani G and McFarlane AS (eds) pp. 357-369. MacMillan Press: London.

SIEGEL JP AND PURI RK. (1991). Interleukin-2 toxicity. *J. Clin. Oncol.*, **9**, 694-704.

VLASVELD LT, RANKIN EM, HEKMAN A, RODENHUIS S, BEIJNEN JH, HILTON AM, DUBBELMAN AC, VYTH-DREESE FA AND MELIEF CJM. (1992). A phase I study of prolonged continuous infusion of low dose recombinant interleukin-2 in melanoma and renal cell cancer. I. Clinical aspects. *Br. J. Cancer*, **65**, 744-500